# The CAIDE dementia risk score indicates elevated cognitive risk in late adulthood: a structural and functional neuroimaging study

**DOI:** 10.1007/s11357-025-01766-8

**Published:** 2025-06-26

**Authors:** Katalin Farkas, Timea Lazar, Melinda Becske, Janos Andras Zsuffa, Viktoria Rosenfeld, Dalida Borbala Berente, Gergo Bolla, Janos Negyesi, Andras Attila Horvath

**Affiliations:** 1 Faculty of Medicine, Semmelweis University, 26 Ulloi Ut, 1085 Budapest, Hungary; 2 School of PhD Studies, Semmelweis University, 26 Ulloi Ut, 1085 Budapest, Hungary; 3 Nyiro Gyula National Institute of Psychiatry and Addictology, Neurocognitive Research Centre, 59-61 Lehel Utca, 1135 Budapest, Hungary; 4 Department of Family Medicine, Semmelweis University, 25 Ulloi Ut, 1085 Budapest, Hungary; 5 Department of Neurosurgery and Neurointervention, Semmelweis University, 57 Amerikai Ut, 1145 Budapest, Hungary; 6 Department of Kinesiology, Hungarian University of Sports Science, 42-48 Alkotas Utca, 1123 Budapest, Hungary; 7 CRU Hungary Ltd, 9-11 Csabai Kapu, 3529 Miskolc, Hungary; 8 Department of Anatomy Histology and Embryology, Semmelweis University, 58 Tuzolto Utca, 1094 Budapest, Hungary; 9 HUN-REN, Research Centre for Natural Sciences, 2 Magyar Tudosok Korutja, 1117 Budapest, Hungary; 10 Faculty of Medicine, University of Lisbon, Alameda da Universidade, 1649-004 Lisbon, Portugal

**Keywords:** CAIDE score, Late adulthood, Cognitive risk, Brain atrophy, Neuropsychology, Functional neuroimaging

## Abstract

While the CAIDE score is an established mid-life risk assessment tool for the longitudinal prediction of dementia, its application as a measure of elevated cognitive risk in late adulthood is unclear. One hundred one healthy individuals (aged > 55) were tested using detailed neuropsychological assessments, as well as structural and functional MRI. Participants were divided into a low-risk group (CAIDE < 7) and a high-risk group (CAIDE > 6). High-risk participants had significantly worse cognitive performance in the Trail-Making Test results (TMT-B: t = -4.03, *p* = 0.0001), and significantly smaller global brain volumes (e.g., brain segmentation volume: t = 2.898, *p* = 0.0485) and regional volumes (e.g., accumbens volumes: t_left_ = 3.928, *p* = 0.036, Cohen’s d = 0.828; t_right_ = 3.151, p_=_ 0.0485, Cohen’s d = 0.656). High-risk participants also showed a trend for reduced functional connectivity in the default mode, salience, and central attention networks. Overall, the CAIDE score might help identify cognitively high-risk individuals in a late adulthood population.

**Supplementary Information:**

The online version contains supplementary material available at 10.1007/s11357-025-01766-8.

## Introduction

Major neurocognitive disorders (NCDs) represent the primary cause of dementia, affecting over 55 million people worldwide [1]. Over the next few decades, the number of people living with dementia is expected to increase significantly because of population aging. Two-thirds of the cases are associated with Alzheimer’s disease (AD), making it the most common cause of NCDs [2]. Dementia is a public health priority, not only because it places a heavy burden on the affected people and their caretakers but also because it places enormous costs on society [1].

Currently, there is no cure for NCDs; therefore, prevention and risk assessment in cognitively unimpaired individuals are crucial. In addition to age, multiple known risk factors associated with AD exist, including modifiable and non-modifiable contributors [3, 4]. Some factors showing a strong positive correlation with the development of dementia act primarily in the midlife period (e.g., ApoE genetic status, along with physical inactivity or cooccurrence of hypertension with obesity and diabetes mellitus) [5]. In contrast, others remain predictive in the late-life period (e.g., a brain hypoperfusion profile associated with chronic heart failure or atherosclerosis, and a profile characterized by high systolic blood pressure and diabetes mellitus) [6]. Protective factors include high education, physical activity, high work complexity, engaging in active leisure activities, and maintaining a healthy cardiovascular status [7]. Over the past few decades, numerous studies have sought to assess the risk of dementia by developing risk scores for various population groups [8]. Validation of risk scores developed for mid-life factors requires long-term follow-ups to monitor the predictive accuracy for the development of dementia [8]. They have been tested in some cases, even in a 40-year longitudinal period [9]. Late-life risk scores have also been introduced (e.g., Late-life Dementia Risk Index, LIBRA score) [10]. Similarly to mid-life risk assessment batteries, the validation of late-life risk scores in dementia development used a short follow-up interval as the primary endpoint. [10, 11]. However, this approach overlooks the fact that functional and structural changes in the brain precede the onset of symptoms in NCDs [12, 13]. An elevated risk for the development of dementia might be captured even in the early prodromal or preclinical stages using some of the surrogate markers of cognitive risk (e.g., fluid and/or neuroimaging biomarkers) [14].

The Cardiovascular Risk Factors, Aging, and Dementia Study (CAIDE) Dementia Risk Score was developed by Kivipelto et al. [15] in a Finnish population to predict 20-year dementia risk among middle-aged individuals. This is the most established and frequently used mid-life risk score for predicting future dementia risk (Supplementary Table 1) [15–28]. The first version of the score contained seven variables: age, education, sex, systolic blood pressure, body mass index (BMI), total cholesterol, and physical activity. The second version of the risk score included one additional variable: APOE status. However, it did not significantly alter the accuracy of the risk score. The CAIDE score was externally validated in a larger population from the Kaiser Permanente Northern California Medical Care Program (KPNC), USA. It was shown to perform well across different ethnicities [9]. The validation study did not include the physical activity variable, as the original research stated that this variable does not significantly correlate with the risk of dementia [9, 15]. Additional variables were included in the study of Exalto et al. [9]. However, they did not improve the predictability of the risk score. There is limited literature available on the CAIDE Dementia Risk Score about baseline cognitive status and brain structure. Interestingly, there are currently no studies on the relationship between CAIDE status and the integrity of functional neural networks. Thus, the predictive potential is not entirely understood at a biological level, and its utility as an indicator of preclinical or prodromal stages still needs to be investigated. In line with this, its application as a late-life risk score has yet to be tested.

The core idea is that CAIDE score could be easily implemented in primary care settings as a late-life cognitive risk score, since it is an easy and rapid risk assessment tool, which could even be automated in an electronic medical record and made available to a clinician at the start of an encounter, could help a busy primary care clinic identify patients more likely to benefit from further and more detailed cognitive screening. To validate this idea, the current study aimed to analyze the association between the CAIDE Dementia Risk Score and baseline cognitive and structural/functional brain status, serving as surrogate markers of cognitive risk, including both neuropsychological tests and neuroimaging data, in elderly, cognitively unimpaired individuals.

## Methods

### Participants

Participants (*n* = 101) of this research project were enrolled from the AlzEpi Cohort Observational Library (ACOL database) of the Neurocognitive Research Centre, Nyírő Gyula National Institute of Psychiatry and Addictology (previously known as National Institute of Mental Health, Neurology and Neurosurgery). The ACOL database is harmonized with other European datasets under the umbrella of the Euro-Fingers Consortium (www.eufingers.com). Every participant was classified as a healthy control individual with normal neurological status and no cognitive decline, as objectively assessed by neuropsychologists. All participants had normal structural brain MRI status, without significant vascular lesions (Fazekas score < 2) or cortical atrophy. Participants were excluded if they had known risk factors of cognitive decline such as vitamin B12 deficiency, hypothyroidism, liver disease, renal insufficiency, clinically meaningful brain lesions (stroke, white matter lesions), demyelinating conditions, hydrocephalus, head injury accompanied by loss of consciousness, use of psychoactive drugs which influence cognitive function, alcohol or substance abuse, schizophrenia, major depression, epilepsy, electroconvulsive therapy, HIV infection, syphilis or preceding central nervous system infections. All research activities took place at the Neurocognitive Research Centre, in accordance with applicable regulations and guidelines. All participants provided written informed consent. The Hungarian Medical Research Council authorized our research (reference number IV/5831 3/2021/EKU).

### Assessment of CAIDE dementia risk score

A modified version (Table 1) of the original CAIDE Dementia Risk Score [15] was utilized in this study. A CAIDE score was calculated for each participant. Points were given for elevated systolic blood pressure or total cholesterol for subjects who had a diagnosis and received or did not receive treatment for hypertension or hyperlipidemia. The cut-off value for total cholesterol was adjusted to 5.2 mmol/L, by Hungarian standards. In the original CAIDE study, physical activity was also included as a variable. However, it was not found to be significantly associated with an increased risk of dementia and was not included in some of the later studies [9, 15, 29]. No systematically collected data were available for physical activity; therefore, this variable was excluded from the risk score used in this study. The maximum value of points for the CAIDE score in our study was 14.

**Table 1:** The original CAIDE score [15] and the modified CAIDE score were used in this study. In line with Hungarian standards, the cut-off value of total cholesterol was adjusted to 5.2 mmol/L

Risk factors (original CAIDE study)	Score	Risk factors (current study)	Score
Age			
< 47 years	0	< 47 years	0
47–53 years	3	47–53 years	3
> 53 years	4	> 53 years	4
Education			
≥ 10 years	0	≥ 10 years	0
7–9 years	2	7–9 years	2
0–6 years	3	0–6 years	3
Sex			
Women	0	Women	0
Men	1	Men	1
Systolic blood pressure			
≤ 140 mm Hg	0	≤ 140 mm Hg	0
> 140 mm Hg	2	> 140 mm Hg	2
Body-mass index			
≤ 30 kg/m^2^	0	≤ 30 kg/m^2^	0
> 30 kg/m^2^	2	> 30 kg/m^2^	2
Total cholesterol			
≤ 6.5 mmol/L	0	≤ 5.2 mmol/L	0
> 6.5 mmol/L	2	> 5.2 mmol/L	2
Physical activity			
Active	0		
Inactive	1		
Total points	15	Total points	14

Physical activity was not included

### Neuropsychological examination

All participants underwent a comprehensive neuropsychological evaluation by a neurologist, neuropsychologist, or trained neuroscientist. Our test battery included the Hungarian version of Addenbrooke’s Cognitive Examination (ACE) [30, 31] for evaluating global cognitive function. ACE allows for the assessment of six cognitive domains: orientation, attention, memory, verbal fluency, language, and visuospatial function. It is a frequently used clinical practice test containing the Mini-Mental State Examination (MMSE)—the Hungarian version of the Rey Auditory Verbal Learning Test (RAVLT) [32]. The RAVLT test is considered a sensitive tool for identifying MCI. Thus, it was one of the measures used to distinguish subjective cognitive impairment from objective cognitive decline [33]. In this test, participants are asked to listen carefully, memorize as many words as possible from the list they will hear, and then repeat every word they remember. The examiner reads 15 words (list A) aloud to the participant, who is then asked to list the words they remembered. This process is repeated five times, resulting in a maximum possible score of 75 for the RAVLTsum5 value. After the fifth repeat, a different list (list B) of fifteen words is read to the participant. Their task is the same: remember and recite as many words from the list as possible. After a thirty-minute waiting period, the participant is asked to list every word from list A that they still remember, resulting in a delayed recall score (RAVLT7). The Trail-Making Test A (TMT-A) was used to evaluate cognitive speed and attention, while the Trail-Making Test B (TMT-B) was chosen to measure cognitive flexibility [34]. When completing the TMT-A, participants are asked to connect numbers from 1 to 25 in ascending order as quickly as possible. In TMT-B, participants must connect numbers in ascending order and letters in alphabetical order, taking turns, e.g., 1-A-2-B-3-C, and so on. To control for the possible adverse effect of anxiety and mood disorders on cognition, the Hungarian version of the Spielberger State and Trait Anxiety Inventory (STAI-S, STAI-T) ([35] and the 13-question version of the Beck Depression Inventory (BDI-13) [36] were applied. To minimize the impact of anxiety and depression on the findings, individuals with a BDI-II score of > 13 or an STAI score of > 45 were not included in this study [37].

### MRI examinations

All participants underwent a brain MRI, which produced a high-resolution anatomical image used for further processing and analysis. A Siemens Magnetom Verio 3 T scanner (Siemens Healthcare, Erlangen, Germany) was used, along with the standard 12-channel head receiver coil. The protocol consisted of T1-weighted 3D MPRAGE (magnetization prepared rapid gradient echo) anatomical imaging (TR (time resolution) = 2.300 ms; TE (echo time) = 3.4 ms; TI = 100 ms; Flip Angle: 12°; Voxel Size: 1.0 × 1.0 × 1.0 mm). The second measurement was a resting-state functional MRI, an EPI-based MRI sequence (TR = 2000 ms; TE = 30 ms; Flip Angle = 79°; Voxel Size = 3 × 3 × 3 mm). The fMRI scan lasted 10 min while participants lay on the table with their eyes closed. The protocol also consisted of a T2-, diffusion-, and a FLAIR-weighted sequence to identify the possible pathological lesions.

### fMRI image preprocessing

We utilized the CONN toolbox [38] for the analysis of resting-state fMRI data. The standard fMRI preprocessing pipeline was implemented, encompassing functional realignment and unwarp, slice-time correction (interleaved for data), detection of outliers (scrubbing based on ART-identified outlier scans), direct segmentation of functional and structural data, normalization (simultaneous segmentation of Gray/White/CSF and MNI normalization), and spatial smoothing. Following pre-processing, an additional quality check was conducted to assess the accuracy of segmentation. A band-pass filter, ranging from 0.008 to 0.09 Hz, was employed to remove physiology-based artifacts and unrelated signal components. Lastly, linear regression was applied to filter out the white matter, CSF signal, and the effects of realignment and scrubbing.

### rs-fMRI metrics

We employed three voxel-based metrics from the CONN Toolbox: Intrinsic Connectivity (ICC), Local Correlation (LCOR), and Fractional Amplitude of Low-Frequency Fluctuations (fALFF). These measures and similar variants have been previously utilized in various neuropsychiatric conditions [39–41]. ICC was leveraged to examine the interconnectedness of distinct brain regions, indicating the strength of a voxel’s connectivity to all other voxels and the number of other voxels connected to a voxel at a specific threshold value [42]. LCOR was used to calculate local connectivity between brain regions, demonstrating the local coherence of each voxel. It represents a voxel’s connectivity with other voxels in neighboring areas, with the degree of adjacency determined by a Gaussian weight function [42]. Our study used the default parameter for the Gaussian function, which was 25 mm. The fALFF measure was utilized to evaluate the magnitude of the signals, reflecting the neural activity of each brain voxel [42].

### Statistical analysis

The direction and strength of the monotonic associations between the CAIDE score and the studied neuropsychological and neuroimaging measures were assessed with Spearman’s correlation coefficient. For between-group comparisons, the Welch independent samples t-test was used. We defined the cut-off CAIDE score for low and high-score individuals in line with the most frequently applied calculation, using the average CAIDE score of the analyzed population [43, 44]. Based on these considerations, a cut-off value for our subjects was 7 (mean CAIDE total = 6.73), which is also the most frequently defined value for a modified CAIDE score [43, 44]. Low-score and high-score groups were identified as follows: individuals with a local CAIDE score of less than 7 were characterized as low-score, while those with a CAIDE score of 7 or greater represented the high-score population. FDR correction for multiple comparisons was applied separately for neuropsychological, structural, and functional MRI data. Statistical significance was determined at *p* < 0.05. To characterize the magnitude of the effects, we reported the effect size values in Cohen’s d. R Studio was used for statistical analysis.

## Results

### Demographic and clinical characteristics

Among the participants, 47 individuals (mean age = 62.7, SD = 7.61) were categorized as low-score, while 54 were classified as high-score individuals (mean age = 70.7, SD = 6.66). The average CAIDE score in the low-score and high-score groups was 5.23 (SD = 0.813) and 8.28 (SD = 0.940), respectively (Table 2).

**Table 2:** The characteristics of the participants

	All participants (*n* = 101)				
		Low-score (CAIDE < 7)	High-score (CAIDE > 6)	statistic (effect size)	p-value
Number of participants		47	54		
Sex	% females	76.6	72.2	chi^2^ = 0.25 (Cramer’s V = 0.05)	*p* = 0.616
Age in years	Mean (SD)	62.7 (7.61)	70.7 (6.66)	t = −5.63 (Cohen’s d = 1.12)	*p* < 0.0001
BMI	Mean (SD)	24.5 (4.09)	27.87 (5.05)	t = −3.59 (Cohen’s d = 0.71)	*p* = 0.0005
Education	% ≥ 10 years	100	100		
Hypertension	% hypertension	12.8	85.2	chi^2^ = 52.8 (Cramer’s V = 0.72)	*p* < 0.0001
Hyperlipidemia	% hyperlipidemia	46.8	85.2	chi^2^ = 16.8 (Cramer’s V = 0.41)	*p* = 0.00004
CAIDE score (range 0–14)	Mean (SD)	5.23 (0.813)	8.28 (0.94)	t = −17.27 (Cohen’s d = 3.45)	*p* < 0.0001

### Neuropsychological performance

**Fig. 1:**
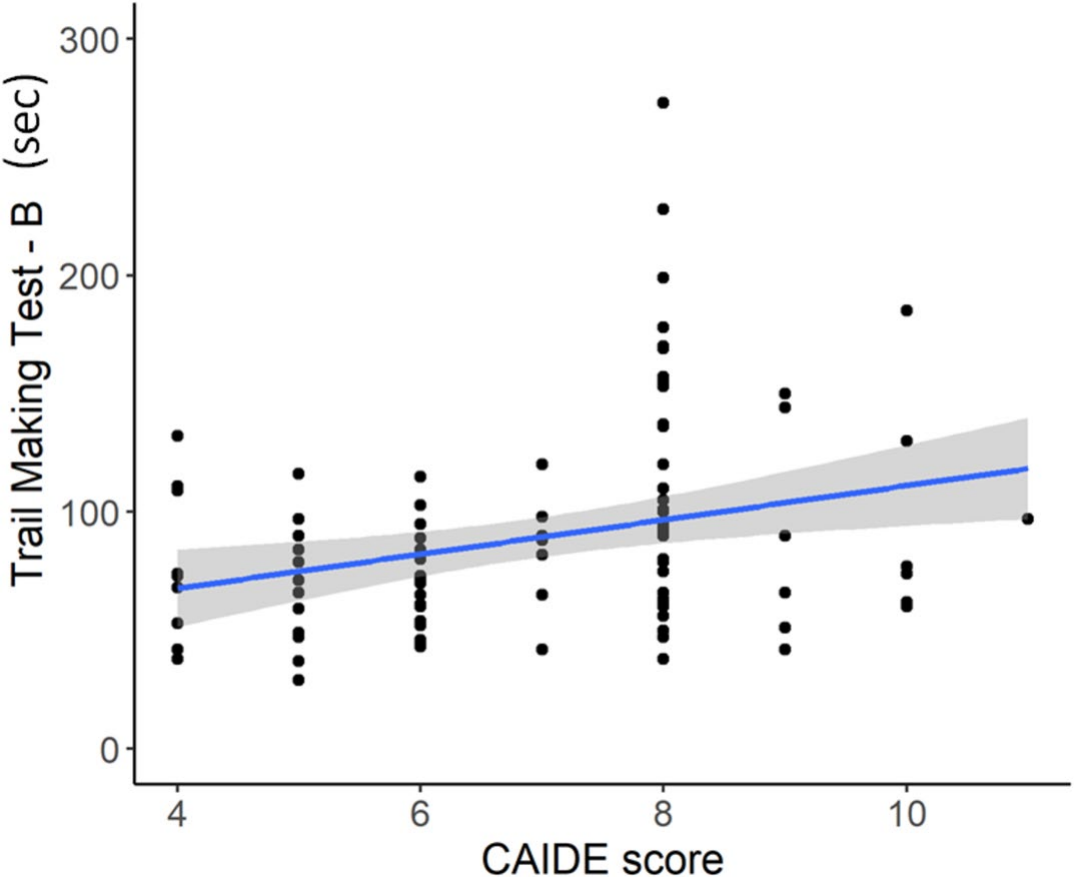
A scatter dot demonstrating the correlation between the CAIDE score and the results of the Trail-Making Test B in seconds (TMT-B). The exact values of the Spearmann-correlation are as follows: r = 0.27 and *p* = 0.0072. The observation reveals that higher CAIDE score associates with poorer cognitive flexibility indicated by elongated TMT-B performance time window

We found a statistically significant between-group difference in Trail-Making-Test B (t = −4.030, *p* = 0.0001, Cohen’s d = −0.768, *low-score* < *high -score*), indicating that the high-score group had difficulties maintaining cognitive flexibility. Differences in the remaining neuropsychological measures were weak and did not survive the Benjamini–Hochberg correction for multiple comparisons (Table 3). The Trail-Making Test B performance was correlated with the CAIDE score (r = 0.270, *p* = 0.0072), indicating that higher CAIDE scores were associated with poorer performance (Fig. 1).

**Table 3:** Between-group differences in neuropsychological performance

Neuropsyscological measure	Low-scoremean (SD)	High-scoremean (SD)	t-value	nominal p-value	Cohen’s d
ACE memory sum	31.36 (3.01)	30.69 (2.46)	1.226	0.2234	0.248
ACE total	93.68 (4.12)	92.89 (4.09)	0.967	0.3358	0.193
VLOM ratio	2.54 (0.30)	2.58 (0.30)	−0.752	0.4537	−0.15
RAVLT_7rate	71.81 (21.56)	66.97 (20.21)	1.152	0.2523	0.232
RAVLT_sum5rate	70.30 (11.23)	65.66 (10.08)	2.157	0.0336	0.437
TMTA	35.69 (12.29)	41.69 (12.65)	−2.385	0.0191	−0.48
TMTB	**71.61 (24.33)**	**102.96 (50.41)**	**−4.030**	0.0001*	**−0.768**
STAI.S	36.77 (11.49)	37.53 (10.73)	−0.325	0.7458	−0.068
STAI.T	43.18 (11.12)	42.08 (8.88)	0.528	0.5991	0.11
BDI.13	5.76 (4.83)	4.84 (4.32)	0.957	0.3415	0.203

P was set as 0.05 after Benjamini–Hochberg correction. * indicates values surviving the correction. *ACE* Addenbrook Cognitive Examination, *VLOM* verbal fluency + language/orientation + memory, *RAVLT* Rey Auditory Verbal Learning Test, *TMT* Trail-Making Test, *STAI* Spielberger State and Trait Anxiety Inventory, *BDI* Beck Depression Inventory

### Structural MRI

**Fig. 2:**
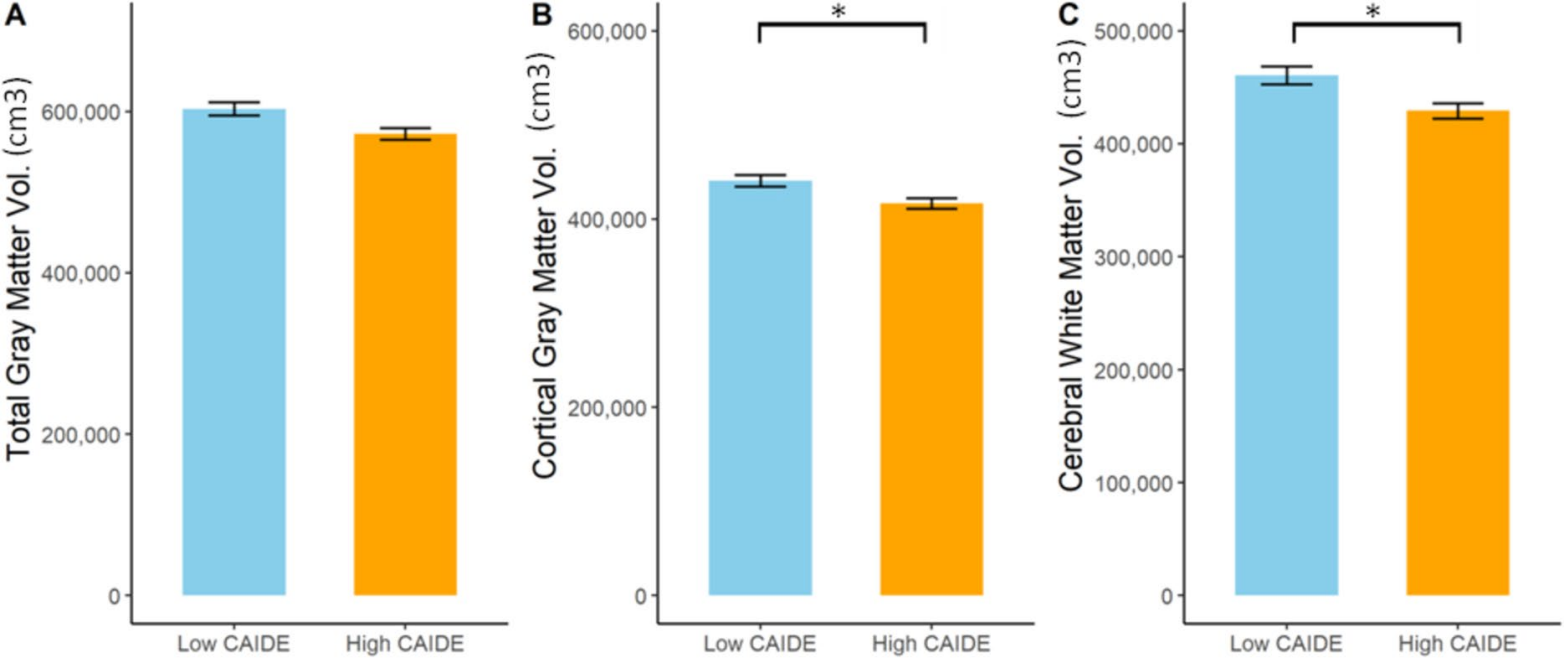
A barplot demonstrating the differences in total volumes (in cubic cm) across participants with low (< 7) and high CAIDE scores (6 <). The exact values are the following: Total gray matter volume (t = 2.76, p_corrected_ = 0.055, Cohen’s d = 0.57), cortical gray matter volume (t = 2.796, p_corrected_ = 0.049, Cohen’s d = 0.579), total cerebral white matter volume (t = 2.938, p_corrected_ = 0.0485, Cohen’s d = 0.611). * indicates significant differences (corrected *p* < 0.05)

Significant differences were found between the low- and high-score groups in terms of global and regional brain volume measures. Brain segmentation volume (BrainSegVol) (t = 2.898, p_corrected_ = 0.0485, Cohen’s d = 0.601), brain segmentation volume without ventricles (BrainSegVolNotVent) (t = 3.067, p_corrected_ = 0.0485, Cohen’s d = 0.637) and the ratio of brain segmentation volume to estimated total intracranial volume (BrainSegVol_to_eTIV) (t = 2.59, p_nominal_ = 0.0112, p_corrected_ = 0.0645, Cohen’s d = 0.54) were smaller in the high-score group. Furthermore, we found reduced total gray matter volume (TotalGrayVol: t = 2.767, p_nominal_ = 0.0069, p_corrected_ = 0.055, Cohen’s d = 0.572), reduced subcortical (SubCortGrayVol: t = 2.627, p_nominal_ = 0.0102, p_corrected_ = 0.0604, Cohen’s d = 0.548), and cortical gray matter volume (CortexVol: t = 2.796, p_nominal_ = 0.0063, *p* = 0.0549, Cohen’s d = 0.579; t_left_ = 2.747, p_nominal_ = 0.0073, p_corrected_ = 0.0549, Cohen’s d = 0.569; t_right_ = 2.829, p_nominal_ = 0.0058, p_corrected_ = 0.053, Cohen’s d = 0.586) in high-score participants. Volumetric reduction was present in the supratentorial area (SupraTentorialVol: t = 2.899, p_nominal_ = 0.0047, p_corrected_ = 0.0485, Cohen’s d = 0.601; SupraTentorialVolNotVent: t = 3.088, p_nominal_ = 0.0027, p_corrected_ = 0.0485, Cohen’s d = 0.642). Moreover, we found lower total cerebral white matter volume in the high-score group (CerebralWhiteMatterVol: t = 2.938, p_nominal_ = 0.0042, p_corrected_ = 0.0485, Cohen’s d = 0.611; t_left_ = 2.708, p_nominal_ = 0.0081, p_corrected_ = 0.0584, Cohen’s d = 0.565; t_right_ = 3.125, p_nominal_ = 0.0024, *p* = 0.0485, Cohen’s d = 0.649). The most characteristic changes are highlighted in Fig. 2.

**Fig. 3:**
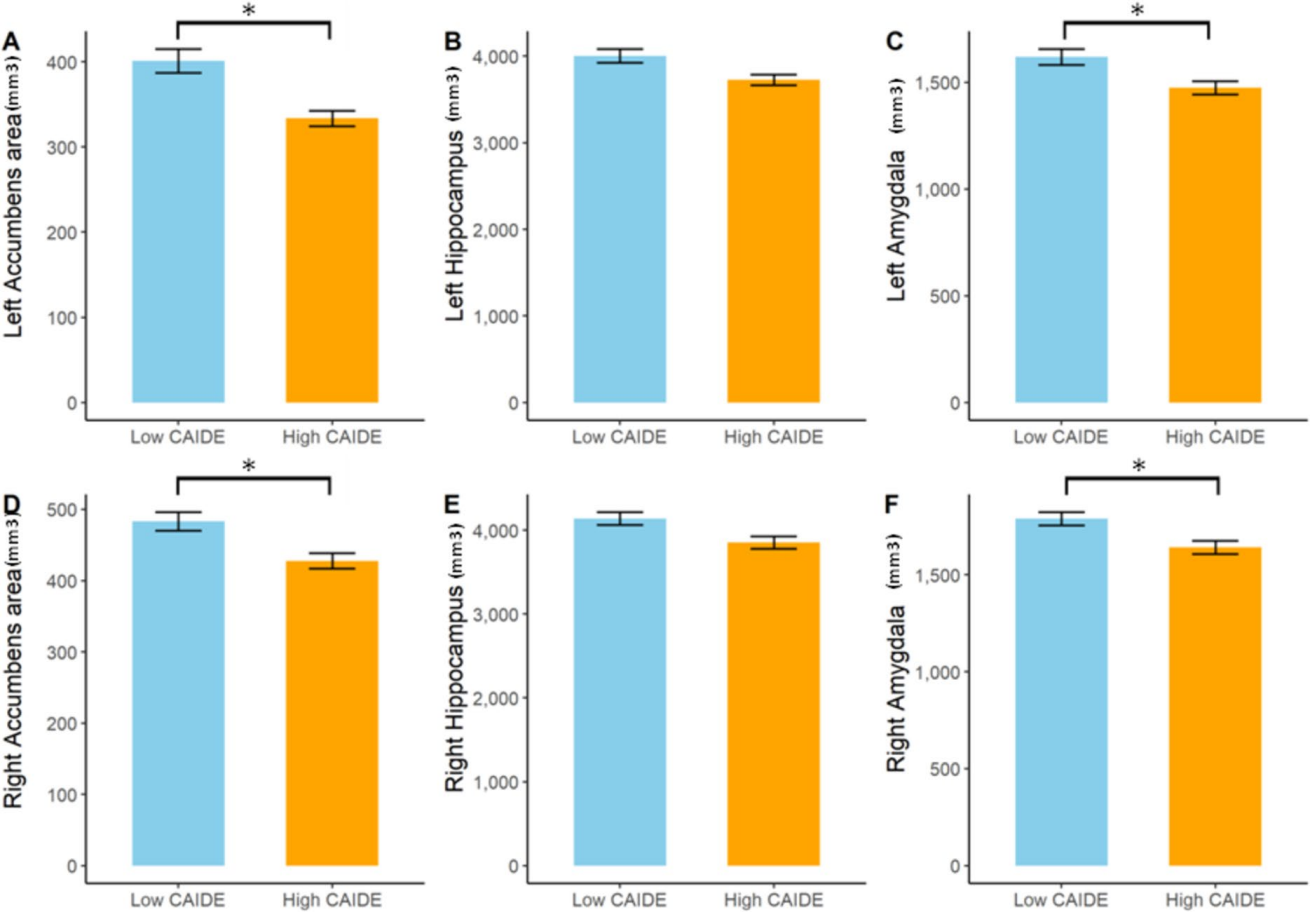
A barplot demonstrating the differences in specific brain areas (in cubic mm) across participants with low (< 7) and high CAIDE scores (6 <). The exact values are the following: Volumes of the left and right accumbens area (t_left_ = 3.928, p_corrected_ = 0.036, Cohen’s d = 0.828; t_right_ = 3.151, p_corrected_ = 0.0485, Cohen’s d = 0.656), volumes of the left and right hippocampus (t_left_ = 2.749, *p* = 0.0549, Cohen’s d = 0.575; t_right_ = 2.624, p_corrected_ = 0.0603, Cohen’s d = 0.541), volumes of the left and right amygdala (t_left_ = 2.927, p_corrected_ = 0.0485, Cohen’s d = 0.61; t_right_ = 2.964, *p* = 0.0485, Cohen’s d = 0.609). * indicates significant differences (corrected *p* < 0.05)

Volumes of the left and right accumbens area (t_left_ = 3.928, p_nominal_ = 0.0002, p_corrected_ = 0.036, Cohen’s d = 0.828; t_right_ = 3.151, p_nominal_ = 0.0022, p_corrected_ = 0.0485, Cohen’s d = 0.656), the left and right hippocampus (t_left_ = 2.749, p_nominal_ = 0.0073, *p* = 0.0549, Cohen’s d = 0.575; t_right_ = 2.624, p_nominal_ = 0.0102, p_corrected_ = 0.0603, Cohen’s d = 0.541), the left and right amygdala (t_left_ = 2.927, p_nominal_ = 0.0044, p_corrected_ = 0.0485, Cohen’s d = 0.61; t_right_ = 2.964, p_nominal_ = 0.0039, *p* = 0.0485, Cohen’s d = 0.609), and the left putamen (t = 2.466, p_nominal_ = 0.0156, p_corrected_ = 0.0821, Cohen’s d = 0.512) were also diminished in the high-score group as indicated in Fig. 3.

Furthermore, regional volumetric differences include reduced volumes in high-score participants in the left and right lateral orbitofrontal (t_left_ = 3.06, p_nominal_ = 0.0029, p_corrected_ = 0.0485, Cohen’s d = 0.636; t_right_ = 2.448, p_nominal_ = 0.0163, p_corrected_ = 0.0835, Cohen’s d = 0.505), right superior frontal (t = 2.842, p_nominal_ = 0.0056, p_corrected_ = 0.0535, Cohen’s d = 0.593), right medial orbitofrontal (t = 2.66, p_nominal_ = 0.0094, p_corrected_ = 0.0596, Cohen’s d = 0.56), left rostral middle frontal (t = 2.656, p_nominal_ = 0.0095, p_corrected_ = 0.0596, Cohen’s d = 0.556), left and right paracentral areas (t_left_ = 2.276, p_nominal_ = 0.0257, p_corrected_ = 0.1122, Cohen’s d = 0.481; t_right_ = 2.971, p_nominal_ = 0.0039, p_corrected_ = 0.0485, Cohen’s d = 0.622), and in the left pars opercularis (t = 2.422, p_nominal_ = 0.0175, p_corrected_ = 0.0851, Cohen’s d = 0.502). Smaller volumes were observed in the left and right middle temporal (t_left_ = 2.916, p_nominal_ = 0.0045, p_corrected_ = 0.0485, Cohen’s d = 0.594; t_right_ = 3.000, p_nominal_ = 0.0035, p_corrected_ = 0.0485, Cohen’s d = 0.622), left inferior temporal (t = 3.011, p_nominal_ = 0.0034, p_corrected_ = 0.0485, Cohen’s d = 0.629), right superior temporal areas (t = 2.941, p_nominal_ = 0.0042, p_corrected_ = 0.0485, Cohen’s d = 0.599) as well as in the left and right inferior parietal areas (t_left_ = 3.385, p_nominal_ = 0.0010, p_corrected_ = 0.0485, Cohen’s d = 0.694; t_right_ = 2.468, p_nominal_ = 0.0155, p_corrected_ = 0.0821, Cohen’s d = 0.500) and also in the right precuneus (t = 2.93, p_nominal_ = 0.0045, p_corrected_ = 0.0485, Cohen’s d = 0.619). Besides these results, diminished cortical volume was observed in the right lateral occipital (t = 2.772, p_nominal_ = 0.0068, p_corrected_ = 0.0549, Cohen’s d = 0.571) and right lingual areas (t = 2.925, p_nominal_ = 0.0044, p_corrected_ = 0.0485, Cohen’s d = 0.605).

In contrast to the observed volumetric reductions, between-group differences in cortical thickness were less prominent. We found decreased cortical thickness in the right paracentral area (t = 2.682, p_nominal_ = 0.0087, p_corrected_ = 0.0588, Cohen’s d = 0.557) and increased cortical thickness in the right rostral middle frontal area (t = −2.696, p_nominal_ = 0.0084, p_corrected_ = 0.0584, Cohen’s d = −0.547) in participants of high-scoreof CAIDE.

**Fig. 4:**
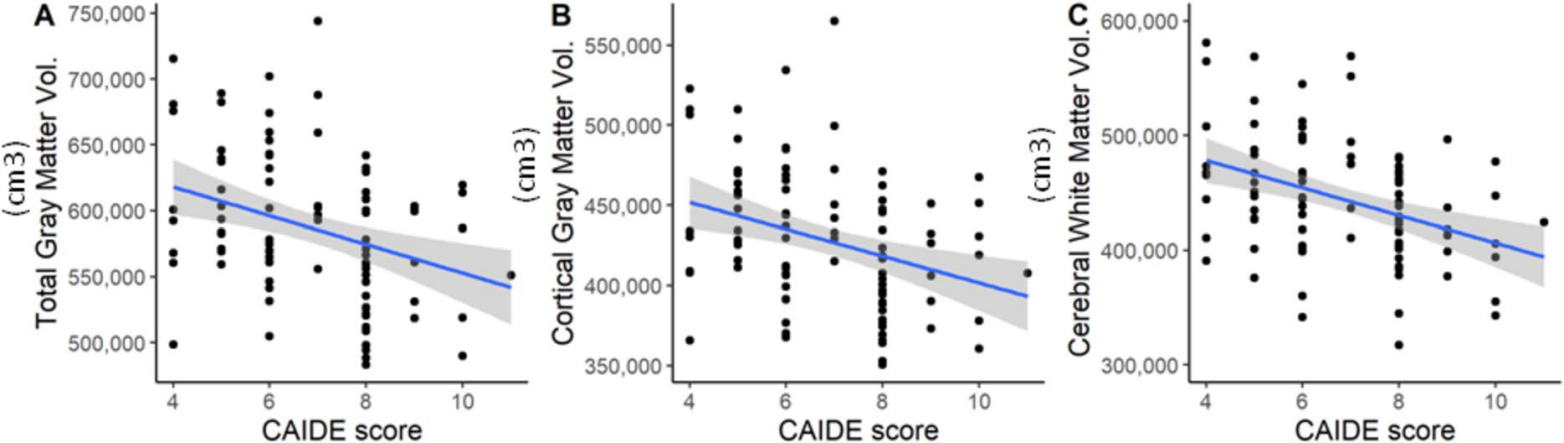
A scatter dot demonstrating the correlation between CAIDE score and total brain volumes (in cubic cm). The exact values of the Spearmann-correlation are the following: Total gray matter volume (r_s_ = −0.332, p_corrected_ = 0.0109), cortical gray matter volume (CortexVol: r_s_ = −0.351, p_corrected_ = 0.0084), total cerebral white matter volume (CerebralWhiteMatterVol: r_s_ = −0.379, p_corrected_ = 0.0051). The observation reveals that higher CAIDE score associates with smaller brain volumes

Apart from the prominent between-group differences, the CAIDE score also correlated negatively with mostly volumetric-structural MRI measures, indicating that higher CAIDE scores were related to lower brain volume (both on global and regional levels) (Fig. 4).

We found statistically significant correlations between the CAIDE score and global volumetric measures such as brain segmentation volume (BrainSegVol: r_s_ = −0.363, p_nominal_ = 0.0003, p_corrected_ = 0.0064, and BrainSegVolNotVent: r_s_ = −0.376, p_nominal_ = 0.0002, p_corrected_ = 0.0051), the ratio of brain segmentation volume to estimated total intracranial volume (r_s_ = −0.215, p_nominal_ = 0.0371, p_corrected_ = 0.1021), total gray matter volume (r_s_ = −0.332, p_nominal_ = 0.0011, p_corrected_ = 0.0109), cortical gray matter volume (CortexVol: r_s_ = −0.351, p_nominal_ = 0.0006, p_nominal_ = 0.0005, p_corrected_ = 0.0084; r_s left_ = −0.347, *p* = 0.0090; r_s right_ = −0.346, p_nominal_ = 0.0006, p_corrected_ = 0.0090), subcortical gray matter volume (r_s_ = −0.316, p_nominal_ = 0.0019, p_corrected_ = 0.0154), supratentorial volume (SupraTentorialVol: r_s_ = −0.375, p_nominal_ = 0.0001, p_corrected_ = 0.0051; SupraTentorialVolNotVent: r_s_ = −0.387, p_nominal_ = 0.0001, p_corrected_ = 0.0051), and total cerebral white matter volume CerebralWhiteMatterVol: r_s_ = −0.379, p_nominal_ = 0.0002, p_corrected_ = 0.0051; r_s left_ = −0.364, p_nominal_ = 0.0003, p_corrected_ = 0.0064; r_s right_ = −0.403, p_nominal_ = *p* < 0.0001, p_corrected_ = 0.0051).

Overall, the CAIDE score was the most strongly related to the volume of the left accumbens area (r_s_ = −0.373, p_nominal_ = 0.0002, p_corrected_ = 0.0051). However, correlations were statistically significant in the case of several other brain regions (Supplementary Table 2). The estimates range between −0.268 and −0.403.

Furthermore, right paracentral thickness (r_s_ = −0.229, p_nominal_ = 0.0262, p_corrected_ = 0.0864) correlated negatively to the CAIDE score, while right rostral middle frontal thickness (r_s_ = 0.285, p_nominal_ = 0.0054, p_corrected_ = 0.0298), and right pars orbitalis thickness (r_s_ = 0.229, p_nominal_ = 0.0264, p_corrected_ = 0.0864) were positively related to it, meaning that higher CAIDE scores were associated with higher cortical thickness in these two areas.

### Functional MRI

**Fig. 5:**
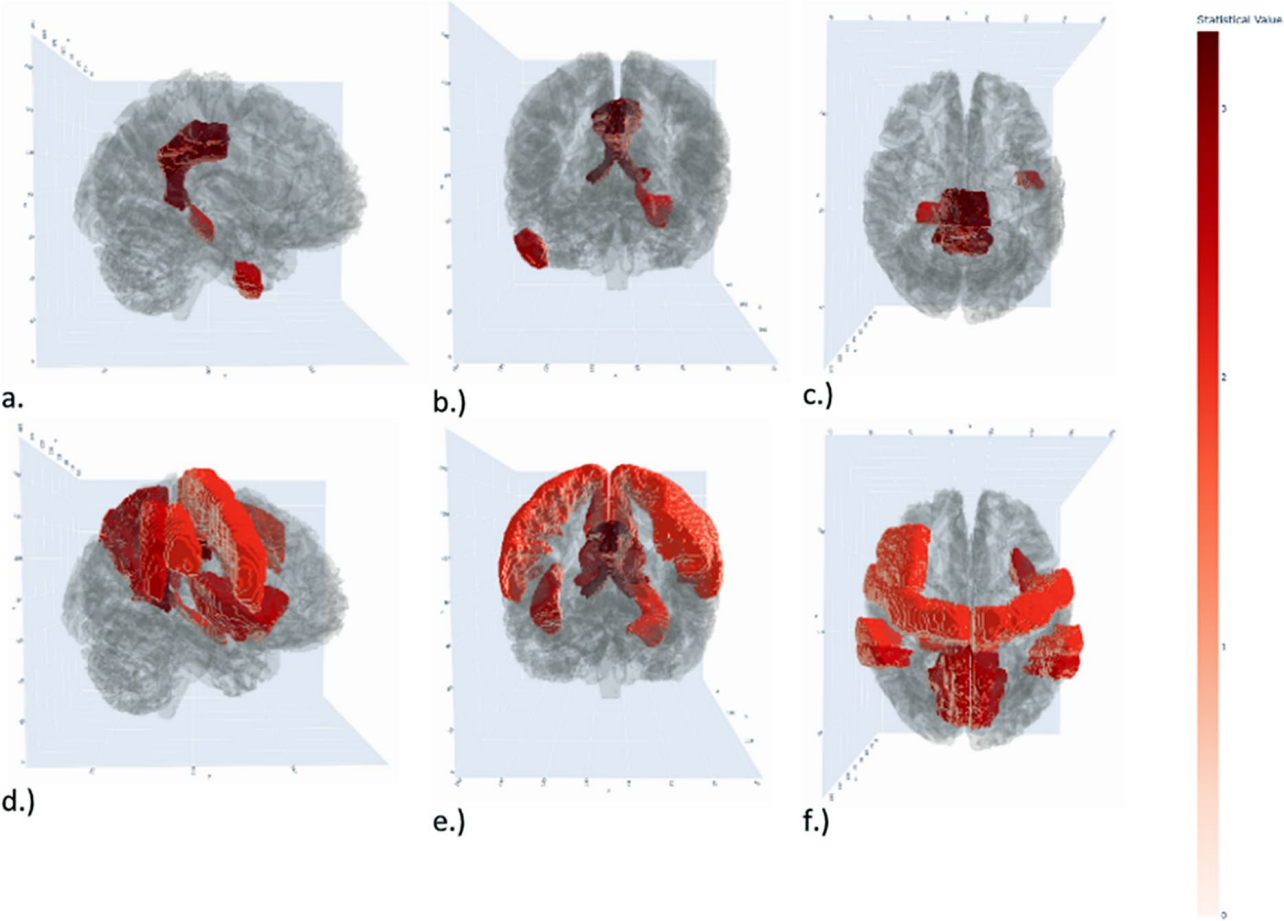
A glass brain demonstrating the t-values for regions from the rs-fMRI analysis: LCOR metric showing Cingulate Gyrus, posterior division, Parahippocampal Gyrus, posterior division Right side and Inferior Temporal Gyrus, anterior division Left side. a.) LCOR metric lateral view, b.) LCOR metric front view, c.) LCOR metric top view. fALFF metric showing Cingulate Gyrus, posterior division, Cingulate Gyrus, posterior division, Precuneous, Precentral Gyrus Left and Right side, Hippocampus Right side, Insular Cortex Left and Right side, Supramarginal Gyrus, anterior and posterior division and the Middle Frontal Gyrus Right d.) fALFF metric lateral view, e.) fALFF metric front view, f.) fALFF metric top view. Key: DMN default mode network; SN salience network; CEN central executive network; LCOR local correlation; fALFF fractional amplitude of low-frequency fluctuation

Functional MRI results (intrinsic connectivity (ICC), local correlation (LCOR), and fractional amplitude of low-frequency fluctuation (fALFF)), in general, indicated a tendency of mostly weaker large-scale and regional connectivity, as well as lower fractional amplitude of low-frequency fluctuation in individuals with a high score of CAIDE Fig. 5). In most cases, a negative relationship was observed between the higher dementia CAIDE risk score and the measured fMRI indices in group comparisons and correlation analyses; however, none of the results survived the Benjamini–Hochberg correction (Supplementary Table 3).

Regarding some crucial regions of the Default Mode Network (DMN), LCOR was weaker in the posterior cingulate gyrus (t = 2.574, p_nominal_ = 0.005, p_corrected_ = 0.4846, Cohen’s d = 0.53) and in the left posterior division of the superior temporal gyrus (t = 2.146, p_nominal_ = 0.0349, p_corrected_ = 0.4846). Similarly, negative correlations were found between the CAIDE score and LCOR values; the strongest correlations were observed with the posterior cingulate gyrus (r_s_ = −0.276, p_nominal_ = 0.0074, p_corrected_ = 0.3220). But at the same time, LCOR was higher in the left anterior division of the inferior temporal gyrus in the high-risk group (t = −2.158, p_nominal_ = 0.0335, p_correcter_ = 0.4846, Cohen’s d = −0.445).

Furthermore, in most regions, fALFF was lower in the high-score group. Regarding the areas of the DMN, fALFF values in the posterior cingulate gyrus (t = 3.214, p_nominal_ = 0.0018, p_corrected_ = 0.4846, Cohen’s d = 0.66), in the precuneus (t = 2.673, p_nominal_ = 0.0089, p_corrected_ = 0.4846, Cohen’s d = 0.551), in the precentral gyrus (t = 2.301, p_nominal_ = 0.0237, p_corrected_ = 0.4846, Cohen’s d = 0.475), as well as in the right hippocampus (t = 2.118, p_nominal_ = 0.0369, p_corrected_ = 0.4846, Cohen’s d = 0.44) were lower in the high-score group. The strongest negative correlation was observed in the posterior cingulate gyrus (r_s_ = −0.2822, p_nominal_ = 0.0061, p_corrected_ = 0.3220). Concerning the SN, lower fALFF was found in the insular cortex (t_left_ = 2.403, p_nominal_ = 0.0183, p_corrected_ = 0.4846, Cohen’s d = 0.499; t_right_ = 2.095, p_nominal_ = 0.0391, p_corrected_ = 0.4846, Cohen’s d = 0.437). In case of regions of the Central Executive Network (CEN), there was a tendency of lower fALFF values in the high-score group in the posterior (t = 2.245, p_nominal_ = 0.0272, p_corrected_ = 0.4846, Cohen’s d = 0.463) and anterior supramarginal gyrus (t = 1.87, p_nominal_ = 0.0648, p_corrected_ = 0.5434, Cohen’s d = 0.382), as well as in the right middle frontal gyrus (t = 1.769, p_nominal_ = 0.080, p_corrected_ = 0.5566, Cohen’s d = 0.364). This tendency was also reflected in a negative correlation between the CAIDE score and fALFF values.

## Discussion

The present study investigated the potential utility of the CAIDE score in cognitively healthy, late adulthood populations to indicate elevated risk for cognitive decline using surrogate biomarkers of cognitive risk. We investigated the relationship between CAIDE dementia risk scores and different mental and brain status measures, including structural and functional neuroimaging. When comparing high- and low-score CAIDE groups in terms of neuropsychological test results, a decreased performance in TMT scores was found among the high-risk score group. Significant differences were found between the structural imaging data of the two groups; both global and regional brain volumes were reduced in the high-score group. While no significant alterations were observed in the functional networks, a trend that indicated decreased functional connectivity in the large-scale neural networks was detected.

Multiple studies have analyzed the predictive value of the mid-life CAIDE score in predicting dementia, including longitudinal changes in brain volumes, cerebrospinal fluid (CSF) profiles, and cognitive status (see Supplementary Table 1) [15–28]. These results consistently report that the mid-life CAIDE score accurately indicates accelerated cortical thinning [45] and the progression of cognitive deterioration [25]. However, the utility of the CAIDE score in detecting cognitive risk in the healthy late-adulthood population is barely estimated, as only two studies [43, 46] have analyzed the association between objective biomarkers of mental status (namely, neuropsychology) and the late-life CAIDE score. While white matter lesions have been assessed in one study [46], additional structural MRI measures, such as brain volumes, were not applied. Interestingly, the association between functional neuroimaging findings and the CAIDE score has been assessed in only one study [47]. Our findings align with these observations; however, our report is the first to systematically investigate the association between late-life CAIDE scores and objective measures of cognitive and brain status, including neuropsychology, structural, and functional neuroimaging.

A study by Ecay-Torres et al. compared the amyloid-tau CSF status, white matter hyperintensities, and cognitive scores of cognitively normal participants with low and high CAIDE scores [46]. The mean age of this cohort was 57.6 years, representing a late middle-age cohort. It was found that high-score participants had significantly poorer executive function, visual perception, and construction abilities. A cross-sectional analysis on a late adulthood population was conducted by Tolea et al. [43] using community-based (230 individuals) and clinical samples (219 individuals). The clinical sample contained data from 41 healthy participants. In both cohorts, participants with a higher modified CAIDE (mCAIDE) score exhibited lower cognitive performance, as indicated by the results of multiple test batteries, including the Montreal Cognitive Assessment, animal naming, Hopkins Verbal Learning Test, and TMT. A limitation of this study, which complicates comparisons with the present findings, is that the community sample was not tested with imaging, CSF, or blood-based biomarkers, so their clinical cognitive and brain status could not be explored. Furthermore, the healthy population was not analyzed independently in the clinical sample. Overall, the overlap between our results and the previous findings is that a higher late-life CAIDE score is strongly associated with poor cognitive performance, even in healthy individuals.

A key result of our observation is the reduced performance in TMT scores among healthy individuals with high CAIDE scores. Previous reports highlighted that a reduction of TMT scores in late adulthood predicts accelerated mobility impairment and increased mortality [48]. Decreased TMT performance was also associated with an elevated cognitive risk profile in multiple experiments. In the study of Oosterman et al., it was associated with higher medial temporal lobe atrophy scores [49]. In the experiment by Terada et al., lower TMT results were consistent with significant hypoperfusion in the bilateral anterior cingulate cortex, bilateral striatum, and bilateral thalamus [50]. It has also been proposed that the reduced TMT performance indicates elevated motoric cognitive risk, reflecting the higher risk for the development of MCI [51]. Importantly, TMT-B was the best single predictor for predicting dementia (AUC 0.89, HR 2.5) in the Gothenburg MCI study [52]. Considering these findings, the reduced performance in TMT scores, particularly regarding higher CAIDE scores, may indicate an increased risk of cognitive decline in our healthy aging cohort. However, further follow-up is required to validate the prognostic power of the proposed observation.

The structural MRI results further support the proposed concept. To our knowledge, our study is the first to analyze the connection between late-life CAIDE scores and structural brain volumes. As a key observation, a higher late-life CAIDE score was followed by global and local cortical thinning. In longitudinal studies, the reduced GM and increased CSF volume were constantly associated with increased dementia incidence [53, 54]. Notably, in the Gothenburg MCI study, the combination of hippocampal volume and TMT-B was the most effective predictor of dementia (HR 25) [52]. In most of the experiments, the atrophy of the medial temporal lobe, insula, hippocampus, and amygdala showed the most substantial predictive value [53, 55]. With the increased number of affected brain regions, the dementia risk is also elevated [53]. The key role of limbic areas is supported by a brain age prediction study, showing that the grey matter intensity around the hippocampus and amygdala drives the difference between predicted and chronological brain age [56]. While the volumetric reduction of the hippocampus and amygdala was the strongest predictor in the Rotterdam study, the predictive value of the striatum has also been highlighted [57]. In other studies, the steepest decline in cognitive functions was primarily related to the thinning of the ventral striatum (e.g., the accumbens area) [58]. In the current study, it is intriguing to postulate that the strong negative correlation between CAIDE scores and the volumes of key brain areas, including the hippocampi, accumbens area, and amygdala, suggests that the high-CAIDE group may have a higher risk of future cognitive decline. According to our interpretation, the mild to moderate correlation values between volumes and CAIDE can also be partially explained by the study characteristics, namely, the represented population does not exhibit severe atrophy due to their clinical and neuropsychological status. As we proposed previously, longitudinal measurements of the cognitive status of the recruited participants are necessary to validate this concept on an individual level.

Functional MRI studies could add essential aspects to the entire concept. The study by Cao et al. is the only one to analyze the link between CAIDE scores and functional MRI findings in a late-middle-aged population from the PREVENT-Dementia study [47]. Notably, global functional connectivity was a significant predictor of CAIDE scores in the entire cohort. On a network level, the connectivity changes between the sensorimotor, cingulo-opercular, frontoparietal, and default mode networks were the most predictive. In our study, reduced connectivity across various brain areas has also been highlighted. However, the differences did not survive the rigorous correction for multiple comparisons. Notably, a clear trend was visible, as functional connectivity was reduced in key large-scale networks, including the CEN, SN, and DMN. Disruption of the DMN is one of the earliest functional changes in the preclinical stage of dementia [59], and reduced antero-posterior connectivity is a characteristic hallmark of accelerated aging [60]. Similar observations have been described in the connectivity pattern of SN [61] and CEN [62].

## Limitations

There is a key limitation in our study. While we systematically investigated healthy individuals based on their clinical profiles, neuropsychological performance, and structural MRI reports, some participants may have had preclinical NCDs. Although we do not know the amyloid status of the participants, our results suggest that the score system itself may help identify individuals with a higher cognitive risk, as indicated by brain imaging and neuropsychological assessments. This knowledge could be implemented in the first stages of the care system without knowing the biological profile of the examined individual. Furthermore, longitudinal data on the possible development of cognitive decline were not available, so the hard endpoint for the development of dementia is missing. Since we use a register-based follow-up system for the recruited participants, the incidence of dementia in the analyzed population will be communicated as a future direction. However, the main aim of the study was to assess the utility of the late-life CAIDE score as an indicator of increased risk for cognitive decline. The applied biomarkers collectively support the elevated risk as surrogate markers, evidenced by reduced performance in traditional neuropsychological metrics, cortical thinning, and decreased functional connectivity in key cognitive neural networks. Another limitation is the overrepresentation of highly educated females in the study population, which represents a weakness of the study compared to population-wide or registry-based observations. Interestingly, this may also be the case, as it enhances the utility of cardiovascular risk components (e.g., cholesterol level) in the CAIDE score for cognitive risk estimation, compared to gender and education factors, since male gender and low education are associated with a higher CAIDE score. The multimodal examination approach, combined with strict statistical analysis and detailed reporting of corrected p-values, full data tables, and effect sizes, represents the strengths of our study.

## Conclusions

In conclusion, assessment of the CAIDE score might help identify cognitively high-risk individuals in a late adulthood population. Since the extraction of the CAIDE score is relatively straightforward in primary care settings, it could serve as a red flag index for elevated dementia risk. As a key suggestion from our findings, the increased CAIDE score in individuals over the age of 55 could help personalize medical decisions to identify those who may require regular cognitive assessments, fluid biomarker testing, and multidomain lifestyle interventions.

## Supplementary Information

Below is the link to the electronic supplementary material.

Supplementary file1 (XLSX 20 KB)

Supplementary file2 (XLSX 39 KB)

Supplementary file3 (XLSX 66 KB)

## Data Availability

All data generated or analysed during this study are included in this published article (and its supplementary information files). The raw imaging data is available upon reasonable request sent to the last author.
